# Assessing the Impact of an Artificial Intelligence-Based Model for Intracranial Aneurysm Detection in CT Angiography on Patient Diagnosis and Outcomes (IDEAL Study)—a protocol for a multicenter, double-blinded randomized controlled trial

**DOI:** 10.1186/s13063-024-08184-9

**Published:** 2024-06-04

**Authors:** Zhao Shi, Bin Hu, Mengjie Lu, Zijian Chen, Manting Zhang, Yizhou Yu, Changsheng Zhou, Jian Zhong, Bingqian Wu, Xueming Zhang, Yongyue Wei, Long Jiang Zhang, on behalf of the China Aneurysm AI Project Group

**Affiliations:** 1 Department of Radiology, Jinling Hospital, Affiliated Hospital of Medical School, Nanjing University, Nanjing, 210002 China; 2 Health Science Center, Ningbo University, Zhejiang, 315211 China; 3 Department of Biostatistics, School of Public Health, Nanjing Medical University, Nanjing, 210002 China; 4 Department of Computer Science, The University of Hong Kong, Hong Kong, China; 5 Jinling Hospital, Nanjing Medical University, Nanjing, 210002 China; 6 Center for Public Health and Epidemic Preparedness & Response, Peking University, Beijing, 100191 China

**Keywords:** Artificial intelligence, Intracranial aneurysms, Randomized controlled trial, Double blinded, Detection, Outcomes

## Abstract

**Background:**

This multicenter, double-blinded, randomized controlled trial (RCT) aims to assess the impact of an artificial intelligence (AI)-based model on the efficacy of intracranial aneurysm detection in CT angiography (CTA) and its influence on patients’ short-term and long-term outcomes.

**Methods:**

*Study* *design*: Prospective, multicenter, double-blinded RCT.

*Settings*: The model was designed for the automatic detection of intracranial aneurysms from original CTA images.

*Participants*: Adult inpatients and outpatients who are scheduled for head CTA scanning.

Randomization groups:

(1) Experimental Group: Head CTA interpreted by radiologists with the assistance of the True-AI-integrated intracranial aneurysm diagnosis strategy (True-AI arm).

(2) Control Group: Head CTA interpreted by radiologists with the assistance of the Sham-AI-integrated intracranial aneurysm diagnosis strategy (Sham-AI arm).

*Randomization*: Block randomization, stratified by center, gender, and age group.

*Primary outcomes:* Coprimary outcomes of superiority in patient-level sensitivity and noninferiority in specificity for the True-AI arm to the Sham-AI arm in intracranial aneurysms.

*Secondary outcomes:* Diagnostic performance for other intracranial lesions, detection rates, workload of CTA interpretation, resource utilization, treatment-related clinical events, aneurysm-related events, quality of life, and cost-effectiveness analysis.

*Blinding:* Study participants and participating radiologists will be blinded to the intervention.

*Sample size*: Based on our pilot study, the patient-level sensitivity is assumed to be 0.65 for the Sham-AI arm and 0.75 for the True-AI arm, with specificities of 0.90 and 0.88, respectively. The prevalence of intracranial aneurysms for patients undergoing head CTA in the hospital is approximately 12%. To establish superiority in sensitivity and noninferiority in specificity with a margin of 5% using a one-sided *α* = 0.025 to ensure that the power of coprimary endpoint testing reached 0.80 and a 5% attrition rate, the sample size was determined to be 6450 in a 1:1 allocation to True-AI or Sham-AI arm.

**Discussion:**

The study will determine the precise impact of the AI system on the detection performance for intracranial aneurysms in a double-blinded design and following the real-world effects on patients’ short-term and long-term outcomes.

**Trial registration:**

This trial has been registered with the NIH, U.S. National Library of Medicine at ClinicalTrials.gov, ID: NCT06118840. Registered 11 November 2023.

**Supplementary Information:**

The online version contains supplementary material available at 10.1186/s13063-024-08184-9.

## Introduction

Artificial intelligence (AI) has had a tremendous influence on the interpretation of medical images [1], such as the diagnosis of intracranial aneurysms in CT angiography (CTA), which is the first-line imaging examination [2–4]. In routine clinical practice, radiologists often misdiagnose intracranial aneurysms due to their small size, complexity of intracranial vasculature [5], and heavy workload [6], which may impose risk for patients with intracranial aneurysms, which can cause nontraumatic subarachnoid hemorrhage (SAH) with a high rate of mortality and disability [7–9]. AI has demonstrated improved reader performance on limited retrospective datasets [10–13], while critics point out that AI systems may be less helpful than retrospective data would suggest and almost all previous AI studies were performed in an open-label design (comparing “AI + readers” vs AI alone or readers alone), which would introduce Hawthorne effect and automation biases [14–16]. There is a lack of high-level evidence for the real-world evaluation of AI systems, especially prospective, real-world, double-blinded randomized controlled trials (RCTs), which are the highest standard evidence in this field.

Moreover, there is still a lack of relevant researches on the impact of AI on the consequent clinical practice and patient outcomes [17, 18]. Accuracy alone is not enough to determine clinical utility because the information gained from diagnostic testing does not have a direct effect on patient outcomes [19]. Recently, Kim and colleagues found that, compared with the AI model, nearly 90% of the aneurysms missed in the clinical radiology report while detected by AI received no further reference; of the aneurysms detected by clinical radiology report, 42.3% underwent further clinical management [20]. Apparently, accurate diagnosis of intracranial aneurysms would alert clinical teams and patients themselves; therefore, it would facilitate patients’ subsequent care downstream in clinical care [21]. To build trust in medical AI systems, demonstrations of impact on clinical outcomes are highly recommended for AI systems, otherwise resulting in widespread doubts about its real effect [16, 22].

Therefore, by introducing Sham-AI as the placebo control (Shi Z, Hu B, Lu MJ, Zhang MT, Yang HT, He B, Ma JY, Hu CF, Lu L, Li S, et al: Propose and validation of a placebo control for AI models in intracranial aneurysms detection: a Multi-centre, Multi-reader, Blinded Crossover Study, unpublished), we designed this prospective, multicenter, double-blinded RCT with two parallel groups and a 1:1 allocation to the True-AI or Sham-AI arm to more rigorously evaluate the hypothesis that a deep-learning-based model for intracranial aneurysm detection in CTA would improve radiologists’ diagnostic performances (the superiority of sensitivity and noninferiority of specificity (a noninferiority margin of 5%) for the intervention group to the control group) and explore patients’ outcomes in the real world to provide the highest level of medical evidence for the clinical deployment of AI systems.

## Methods/design

### Study design

This is a prospective, parallel-group, multicenter, double-blind RCT to investigate the impact of a deep-learning-based computer-aided diagnosis strategy for intracranial aneurysms on the diagnostic performances of radiologists and short-term and long-term outcomes of adult inpatients and outpatients between True-AI and Sham-AI group in a real-world setting. By implementing True-AI and Sham-AI and utilizing randomization, the double-blinded approach is employed to neutralize the subjective influence of participation in an AI trial on diagnostic performance.

**Fig. 1:**
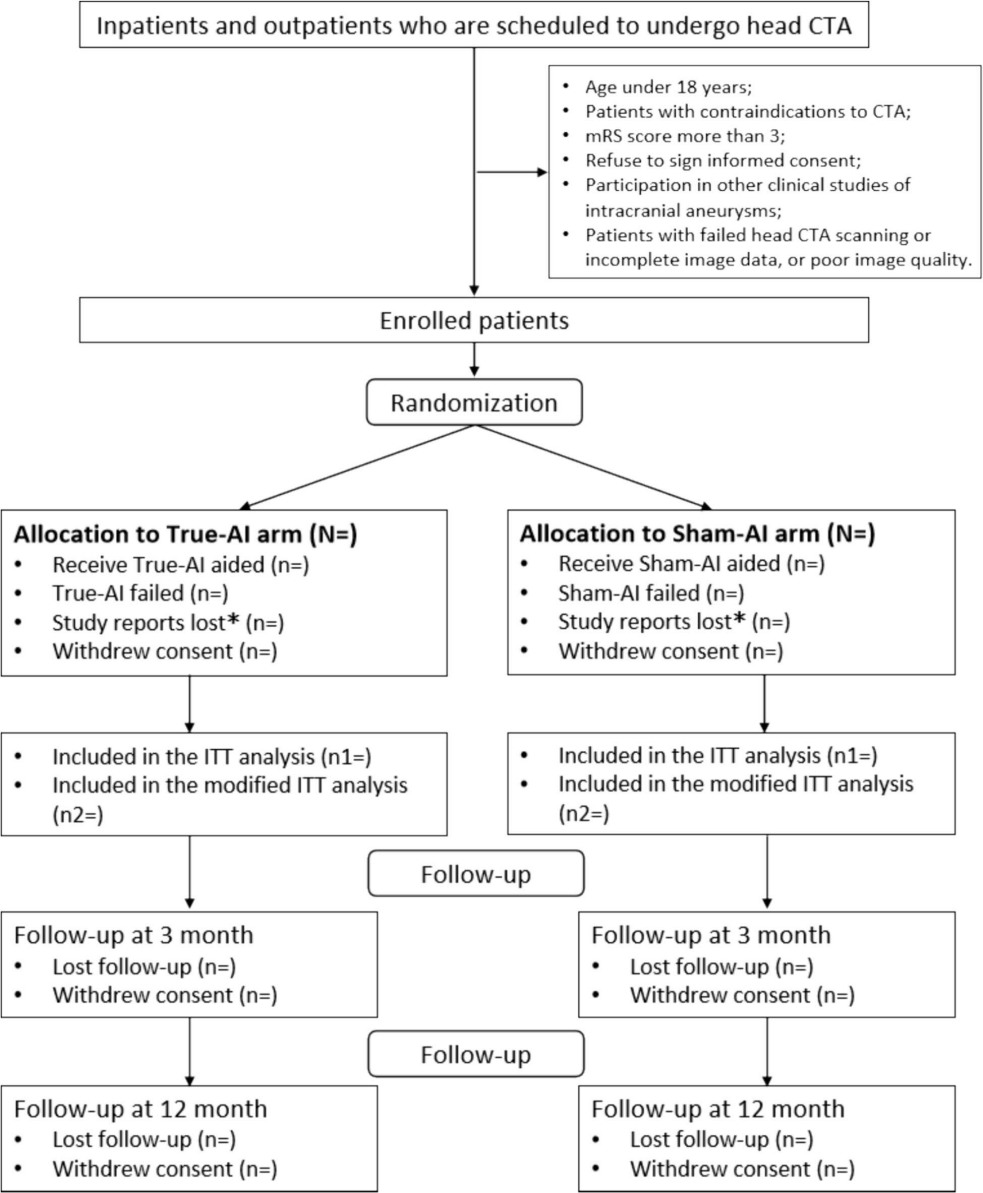
Randomization and follow-up of the patients. *Study reports lost is defined as those whose CTA exams were accidentally interpreted by other radiologists not involved in the trial. AI = artificial intelligence, CTA = CT angiography, mRS = Modified Rankin Scale

The study is being conducted in 25 large-scale, tertiary care hospitals located in 10 provinces across China. Participants will be recruited and randomized into either the experimental or control groups in a 1:1 ratio in each site. In the experimental group, head CTA images will be interpreted by radiologists with True-AI-integrated intracranial aneurysm diagnosis strategy (True-AI arm), which had a patient-level sensitivity of 0.93 in the validation dataset (Shi Z, Hu B, Lu MJ, Zhang MT, Yang HT, He B, Ma JY, Hu CF, Lu L, Li S, et al: Propose and validation of a placebo control for AI models in intracranial aneurysms detection: a Multi-centre, Multi-reader, Blinded Crossover Study, unpublished). In the control group, CTA will be interpreted by radiologists with a Sham-AI-integrated intracranial aneurysm diagnosis strategy (Sham-AI arm), which had a patient-level sensitivity of 0.02 in the same validation dataset, which is close to zero and would not help radiologists detect any aneurysms while expose the radiologists to the same incidental effects of the Standard-AI (Shi Z, Hu B, Lu MJ, Zhang MT, Yang HT, He B, Ma JY, Hu CF, Lu L, Li S, et al: Propose and validation of a placebo control for AI models in intracranial aneurysms detection: a Multi-centre, Multi-reader, Blinded Crossover Study, unpublished) [23]. The study design is illustrated in Fig. 1. This study protocol is reported according to the Standard Protocol Items: Recommendations for Interventional Trials-Artificial Intelligence (SPIRIT-AI) [24]. The SPIRIT-AI Checklist is provided in the Supplementary materials. Table 1 shows the SPIRIT-AI schedule for patient enrollment, intervention, and assessment.

**Table 1:** The SPIRIT-AI schedule of enrollment, interventions, and assessments

	STUDY PERIOD				
	Enrollment	Allocation	Assessments	3-month Follow-up	12-month Follow-up
TIMEPOINT	t1	t2	t3	t4	t5
ENROLLMENT					
Inclusion and exclusion criteria assessment	X				
Informed consent	X				
Allocation to participating radiologists	X				
CTA examination	X				
Randomization	X				
Baseline information collection	X				
INTERVENTION:					
True-AI-integrated diagnosis		X			
Sham-AI-integrate diagnosis		X			
ASSESSMENTS:					
Groundtruth diagnosis			X		
Diagnostic performance assessment			X		
Work load			X		
Resource use				X	X
Treatment-related clinical events				X	X
Life quality evaluation				X	X
Outcomes of aneurysm-related events				X	X
Cost-effectiveness analysis				X	X

*AI* artificial intelligence, *CTA* CT angiography, *MRA* magnetic resonance angiography, *SPIRIT-AI* standard protocol items: recommendations for interventional trials-artificial intelligence

### Participating center qualification

The 25 medical centers are located in 7 different geographical regions of China (North China, Northeast China, East China, Central China, South China, Southwest China, Northwest China). Each center is a tertiary care hospital and has experience in the diagnosis of intracranial aneurysms, with an average of 250 patients undergoing head CTA examination each month. Thus, the cohort can adequately represent the population at the regional and national levels. These centers are needed to shut down other AI-based intracranial aneurysm detection models until patient enrollment is completed.

### Participants and recruitment

Participants who fulfil the following criteria will be eligible:

Inclusion criteria:

- Adult inpatients and outpatients who are scheduled for head CTA scanning.

Exclusion criteria:

- Age under 18 years;
- Patients with contraindications to CTA;
- Modified Rankin Scale (mRS) score > 3;
- Refusal to sign informed consent;
- Participation in other clinical studies of intracranial aneurysms;
- Patients with failed head CTA scanning or incomplete image data or poor image quality.

### Interventions

#### Procedures

**Fig. 2:**
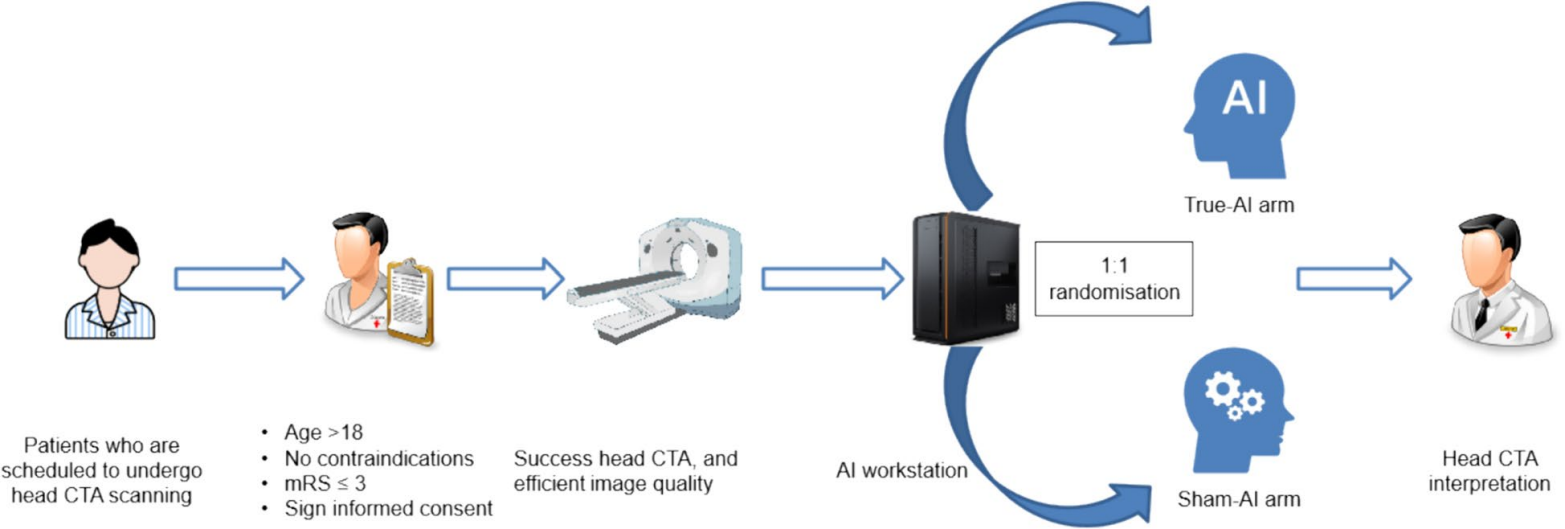
Procedures of patient enrollment and randomization. AI = artificial intelligence, CTA = CT angiography, mRS = Modified Rankin Scale

Eligible patients will be invited to participate in a clinical trial involving the use of an AI-integrated intracranial aneurysm detection strategy by a local staff member, with the option to opt-out if they wish, and will undergo a conventional path without AI assistance (not included in the study population). Written informed consent will be obtained from all participants who agree and wish to take part in the study. Details of case recruitment and study withdrawal are provided in the Supplementary Appendix. Directly after a participant has undergone head CTA scan, the CTA image series will be automatically caught by an onsite hospital-based workstation (DeepWise Aneurysm Aided Detection Software, v1.0.0.2, DeepWise, Beijing, China). Subsequently, the case will be randomized and allocated to either the intervention (True-AI) or control (Sham-AI) arm in a 1:1 ratio, with a pseudorandom number assigned within the workstation. The Sham-AI model can effectively mimic the True-AI model and does not assist doctors in detecting any aneurysms, essentially acting as a placebo (Fig. 2). CTA will be interpreted by radiologists with the assistance of either True-AI or Sham-AI, as part of the study.

The AI-processed image series provided suggestions for suspected intracranial aneurysms, and the radiologists could accept or reject the suggestions according to their own judgment. Both True-AI and Sham-AI can be displayed in an identical human-AI interaction platform.

Each center includes at least two participating radiologists, junior radiologists (usually attending radiologists) writing the initial report, and senior radiologists reviewing and releasing the report, which is the standard of care in China and the radiology community [25]. The participating radiologists need to meet the predefined requirements and been trained for diagnosis of intracranial aneurysms. They will assess the quality of images and data availability. In both study groups, the junior radiologists will interpret CTA images firstly without AI assistance; if aneurysms are diagnosed by junior radiologist, he/she is required to input the three-dimensional coordination of each diagnosed aneurysm in the study website.

**Fig. 3:**
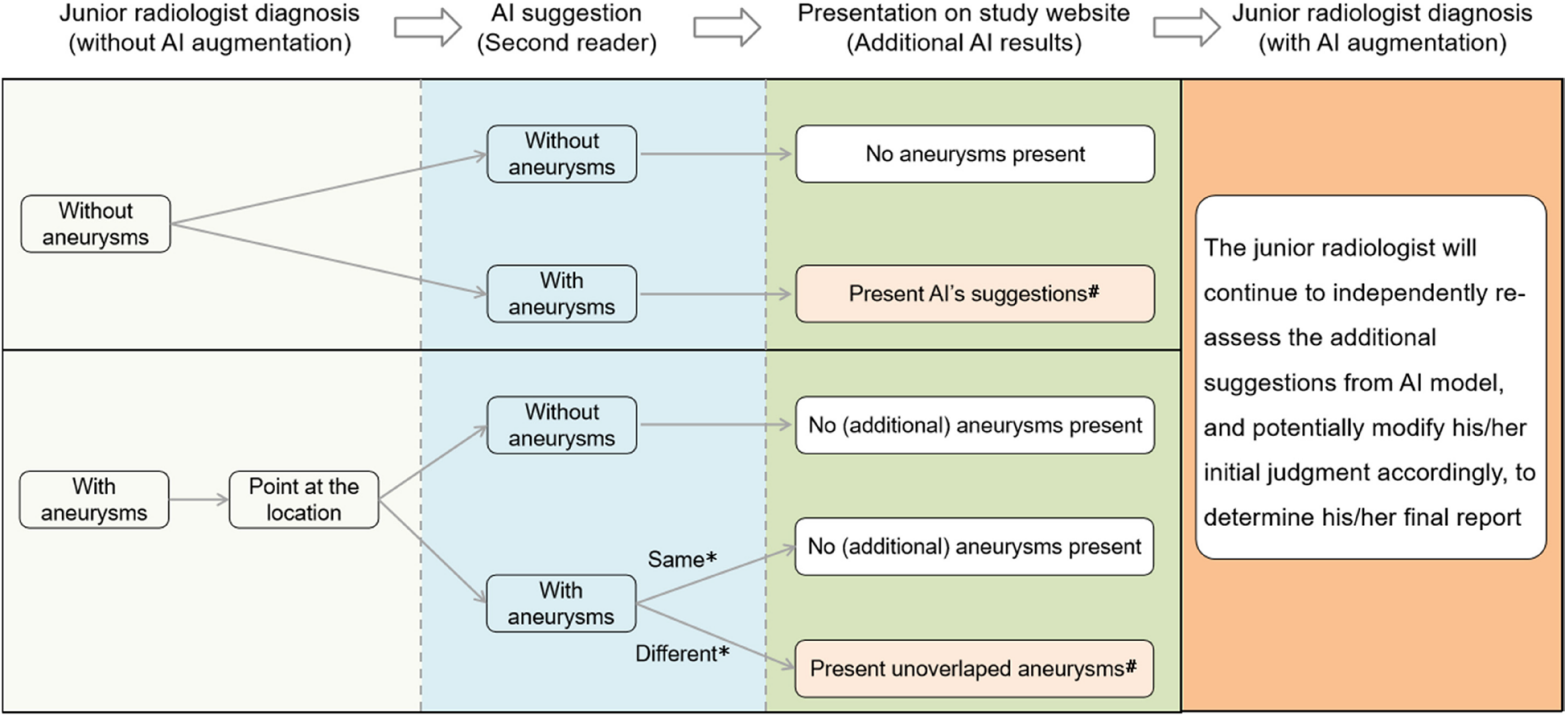
Schematic diagram showing how AI suggestions will be presented according to the diagnosis of junior radiologists. The workstation will judge whether there is overlap between AI suggestions and the initial diagnosis of the junior radiologists; if the two results are identical (same), no additional aneurysms will be presented on the study website; if there are no overlapped with AI suggestions (different), the additional aneurysms will be presented in the workstation. ^#^The study website will only present additional aneurysms by AI models, and they would be true-positive aneurysms or false-positive aneurysms

Then the AI system act as a second reader, and will judge whether the radiologist’s diagnosis is overlapped with the AI suggestions according to the three-dimensional coordination, and only present AI suggestions that are not overlapped with radiologist’s diagnosis on the study website (the radiologist will not have access to the original AI predictions except for the presenting results). The junior radiologist will continue to independently re-assess the additional suggestions, and potentially modify his/her initial judgment accordingly, to determine his/her final report (Fig. 3). The senior radiologist has access to the first reader’s assessment and will review and release the official radiological report. They can assign cases to a consensus meeting when the radiologists find a case difficult or equivocal, where the case will be discussed at their local site for a final decision. After the diagnosis is made, the patients will be treated according to national and institutional guidelines. The requirements for participation, adherence, and protocol deviations and study monitoring are provided in the Supplementary Appendix.

#### Description of the investigational product

The investigational product is a deep-learning-based intracranial aneurysm detection model: True-AI model and Sham-AI model. For both models, the full-resolution Digital Imaging and Communications in Medicine (DICOM) images are directly input without any preprocessing or rescaling. The images will be displayed in a separate web-based platform (Fig. S1 in the Supplementary Appendix).

True-AI model was developed in 16,422 CTA examinations and had a patient-level sensitivity, lesion-level sensitivity and specificity of 0.93, 0.87, and 0.79 in an independent validation dataset of 1810 cases, respectively. The Sham-AI model was developed in the same dataset with a different strategy. It was designed to have a sensitivity close to zero and a similar specificity to True-AI, so that it would not help radiologists detect additional aneurysms and expose the radiologists to the same incidental effects of the Standard-AI at the same time. Finally, Sham-AI had a patient-level sensitivity, lesion-level sensitivity, and specificity of 0.02, 0.01, and 0.80, respectively, in the same validation dataset (Shi Z, Hu B, Lu MJ, Zhang MT, Yang HT, He B, Ma JY, Hu CF, Lu L, Li S, et al: Propose and validation of a placebo control for AI models in intracranial aneurysms detection: a Multi-centre, Multi-reader, Blinded Crossover Study, unpublished). We also have evaluated the influence of Sham-AI on radiologists’ diagnosis of intracranial aneurysms in a crossover, blinded diagnostic study in 28 radiologists from 7 geographically different hospitals across China, and find that radiologists had noninferior sensitivity and specificity with the aid of Sham-AI to that of reader-alone (noninferiority margin of 5%) (Shi Z, Hu B, Lu MJ, Zhang MT, Yang HT, He B, Ma JY, Hu CF, Lu L, Li S, et al: Propose and validation of a placebo control for AI models in intracranial aneurysms detection: a Multi-centre, Multi-reader, Blinded Crossover Study, unpublished). The architecture of the True-AI and Sham-AI models is shown in Fig. S2 in the Supplementary Appendix.

A dedicated medical workstation (DeepWise Aneurysm Aided Detection Software, v1.0.0.2) is designed to demonstrate the DICOM images. The workstation will only present three-dimensional coordination for unoverlapped aneurysms on the study website.

### Eligibility and baseline assessment

For each included participant, the following baseline characteristics will be collected:

1. Demographic information: gender, age, weight (kg), height (cm), education, nationality, registered residence (rural or urban), permanent address;
2. Clinical information: Chief complaint, symptoms, indications, signs, baseline mRS;
3. Medical history: History of intracranial aneurysms, history of head surgery, family history of SAH and intracranial aneurysms, comorbidities such as hypertension, diabetes mellitus, and cardiovascular disease;
4. Lifestyle habits: Smoking, drinking, sleep pattern, exercise, occupation;
5. Life quality evaluation: EuroQol 5-Dimensional, 5-Level (EQ-5D-5L) scores, sleep and psychosocial indexes (Shift Work Assessment, Pittsburgh Sleep Quality Index (PSQI), Patient Health Questionnaire-9 (PHQ-9), Hospital Anxiety and Depression Scale (HADS), Short-Form 36 Health Survey (SF-36);
6. Information from the head CTA and report: Radiology report of head CTA, details on the number, location, size, and morphology of aneurysms, time taken for interpretation, PHASES score [26], and ELAPSS score [27] for unruptured and untreated aneurysms;
7. Others: AI interpretation results, time of Workstation manipulation.

### Reference standard establishment

The reference standard for all CTA examinations will be determined by the Core Image Center, consisting of board-certified neuroradiologists and neurointerventional physicians at 5 large academic tertiary care hospitals with 6–15 years of working experience. Their responsibilities include determining the presence of aneurysm (location and size), intracranial arterial stenosis (≥ 50%) (location and degree of stenosis), occlusion (location), and the presence of intracranial tumors (yes/no). The physicians have access to all of the DICOM series, original reports, clinical history, and previous and follow-up examinations during interpretation and digital subtraction angiograms (DSA), if available, to establish the best possible reference standard for the labels. Each case was labeled and independently checked by two physicians. If the results were consistent, the annotation was adopted. Any disagreement was solved by discussion and consensus reading to review and check the discrepancy and make the final ground truth.

### Randomization

CTA examination will be automatically randomized within the workstation and allocated to either the intervention (True-AI) or control (Sham-AI) arm in a 1:1 ratio. Stratified by center, gender, and age (≤ 54 years or > 54 years) [28] with a combination of block sizes, the randomization sequence will be generated using a computer-generated random numerical series by an independent statistician. The original sequence will be stored in the randomization system database within the AI workstation at each site. If a subject fulfills the enrolment criteria, the enrolled cases will be assigned with a sequence and allocated to one of the groups. The sequence will not be accessible to investigators or study coordinators.

### Blinding

Study participants, local staff members obtaining patients’ consent and radiographers acquiring head CTA exams will be blinded to the randomization process, as it is automatically performed after the examination has been acquired. For radiologists interpreting head CTA exams, because junior radiologists read it firstly without AI augmentation, and AI act as a second reader and only present additional aneurysms according to junior radiologists’ initial diagnosis (Fig. 3), it is challenging for them to distinguish True-AI or Sham-AI in daily practice. Besides, randomization is conducted within the workstation automatically, resembling a “black box”, and participants have no access to the process. Finally, by ensuring that the users of a decision support system feel accountable for their own decisions can also help decrease automation bias [29]. For senior radiologists, they will review and check reports from junior radiologists and have access to the same presentation as the junior radiologists; therefore, they are also blinded to AI distribution.

Bang’s blinding index will be used to evaluate the quality of blinding for the radiologists during the trial [30].

### Plan and methods of follow-up

The participants will be contacted by the WeChat Mini Program or telephone at 3 months and 12 months by a trained team specialized in follow-up. To promote participant retention and complete high-quality follow-up, regular communication with the patients will be conducted. The local staff members will establish effective channels in the invitation by WeChat Mini Program. For nonresponders, the staff will try to contact the participant again 3 days later. The participants’ visit and evaluation schedule is shown in Table 1.

### Outcomes

#### Primary outcomes

When the enrollment is finished, the true-positive, false-positive, true-negative, and false-negative diagnosed aneurysms will be derived from the released reports against the reference standards. The primary outcome measures are coprimary endpoints of superiority in patient-level sensitivity and noninferiority in specificity (with a noninferiority margin of 5% [31]) for the True-AI arm to the Sham-AI arm in intracranial aneurysms. The patient-level sensitivity is defined as the proportion of patients with true-positive diagnosed aneurysms among patients with positive reference standards. The specificity is defined as the proportion of patients with true-negative aneurysms among patients with negative reference standards.

#### Secondary outcomes (Table S1)

- Differences of other diagnostic performance metrics for intracranial aneurysms between True-AI and Sham-AI group, including accuracy, lesion-level sensitivity, positive predictive value (PPV), and negative predictive value (NPV).
- Differences of the diagnostic performances for other intracranial lesions between True-AI and Sham-AI group, including patient-level sensitivity, specificity, accuracy, PPV, and NPV for intracranial arterial stenosis (≥ 50%), occlusion, and intracranial tumors.
- Differences of the detection rates of intracranial lesions according to radiology reports between True-AI and Sham-AI group, including intracranial aneurysms, intracranial arterial stenosis (≥ 50%), occlusion, and intracranial tumors.
- Differences of the workload of head CTA interpretation between True-AI and Sham-AI group, including the time (seconds) of interpreting head CTA images and the number of consensus meetings (times).
- Differences in the proportion of participants with resource use between True-AI and Sham-AI group, including the number of care encounters (in person) during follow-up, the number of care encounters (in person) for aneurysms during follow-up, and the total number of cerebral artery tests (including DSA, CTA, MRA, and high-resolution vessel wall MR imaging) at the 3-month and 12-month follow-ups.
- Differences in the proportion of participants with treatment-related clinical events between True-AI and Sham-AI group including clinical follow-up, hospitalization (number of subsequent hospitalizations, number of hospitalization for intracranial aneurysms, in-hospital mortality rate, morbidity with modified RS ≥ 3 due to intracranial hemorrhage or treatment, length of hospital stay), patients undergoing DSA (detection rate of intracranial aneurysms among DSAs, detection rate of no abnormality among DSAs), patients with different methods for aneurysm treatment (conservative/coil/clip/others), patients with aneurysm treatment-related complications (intraoperative rupture, death, stroke, etc.), patients with recurrence or residual intracranial aneurysm after surgery at the 3-month and 12-month follow-up.
- Differences of life quality evaluation between True-AI and Sham-AI group including EQ-5D-5L scores, sleep and psychosocial indexes (shift work assessment, PSQI, PHQ-9, HADS, SF-36, mRS) at both the 3-month and 12-month follow-up assessments.
- Differences in the proportion of participants with outcomes of aneurysm-related events between True-AI and Sham-AI group including all-cause mortality, mortality of aneurysm rupture, aneurysm growth, aneurysm rupture, SAH, and stroke (hemorrhagic stroke, ischemic stroke) at the 3-month and 12-month follow-ups.
- Differences in the incremental cost-effectiveness ratio (ICER) between True-AI and Sham-AI group at 3-month and 12-month follow-up. We will calculate quality-adjusted life years (QALYs) by using the EQ-5D-5L life quality questionnaire at the 3-month and 12-month follow-up for the True-AI and Sham-AI arms. Healthcare costs per patient will be calculated as the sum of inpatient, outpatient, testing, and pharmaceutical costs during the follow-up period for the two arms. We will divide the difference in healthcare costs by the difference in QALYs to calculate the ICER of the True-AI arm compared to the Sham-AI arm. To determine whether the True-AI arm is cost-effective, we will use cost-effectiveness thresholds with an ICER < $38,070 per QALY gained will be considered cost-effective (Highly cost-effective was defined as an ICER less than 1 time the per capita gross domestic product (GDP) in China; cost-effective, an ICER of 1 to 3 times the per capita GDP; and not cost-effective, an ICER greater than 3 times the per capita GDP [32]. The per capita GDP in China in 2023 was US $ 12,690). We will conduct deterministic sensitivity analysis on all QALYs and cost parameters, typically by one-way or two-way sensitivity analyses. One-way sensitivity analysis involves systematically varying individual parameters through plausible values while holding all other values constant and assessing the impact of these individual variations, while two-way sensitivity analysis involves varying two parameters simultaneously. Our sensitivity analysis will address the uncertainty in the estimation of QALYs and cost parameters and assess its impact on cost-effectiveness results.

### Ethical safety outcomes

Rates of intracranial aneurysms, intracranial arterial stenosis (≥ 50%), occlusion, and intracranial tumors from the final radiology reports will be calculated during the trial and compared with those for the previous 3 months. A reduction in positivity rates of no more than 5% during the trial period will be considered to meet the safety outcome; otherwise, the study will halt to find the reason at the site.

We will further evaluate the false-positive findings that require unnecessary follow-up tests or treatment and extra radiologists’ ruling out time, as well as the false-negative findings that may cause catastrophic consequences.

### Exploration of AI for radiologists’ training

Dynamic changes in the sensitivity and specificity of intracranial aneurysm diagnosis will be tracked from the first 7 days after the study begins through to the last 7 days before the study ends across all centers.

### Safety evaluation

Participants will not be exposed to the investigational product (AI software) but only their head CTA exams. The interventions in this study will not add additional risks to participants compared to routine head CTA interpretation practice. Therefore, adverse events caused by the investigational product are not applicable in this study.

However, if a patient experiences an unexpected adverse event unrelated to the intervention and requires disclosure of study assignment information, unblinding can be performed by statistician with the admission of the PI. Unexpected adverse events unrelated to the intervention will be evaluated according to the Common Terminology Criteria for Adverse Events [33]. The time of occurrence, expiration, interventions, and treatments will be recorded.

### Data analysis

#### Sample size calculation

The success of primary analysis requires both the superiority of sensitivity and noninferiority of specificity (with a noninferiority margin of 5% [31]) of the True-AI arm compared to the Sham-AI. Sample size calculations were done with PASS (v21.0.3).

Sample size calculations for superiority of sensitivity: Using a one-sided *α* level of 0.025 and a power of 0.80, we estimated the sensitivity to be 0.65 in the Sham-AI-integrated intracranial aneurysms diagnosis strategy, as opposed to 0.75 in the True-AI-integrated intracranial aneurysms diagnosis strategy according to our previous study (Shi Z, Hu B, Lu MJ, Zhang MT, Yang HT, He B, Ma JY, Hu CF, Lu L, Li S, et al: Propose and validation of a placebo control for AI models in intracranial aneurysms detection: a Multi-centre, Multi-reader, Blinded Crossover Study, unpublished). With a 10% increase in sensitivity from Sham-AI to True-AI and accounting for a 5% attrition rate due to that participants withdraw consent [34], we estimated that a sample size of 694 patients with intracranial aneurysms is required to achieve the desired statistical power. Based on our previous experience, assuming a prevalence of intracranial aneurysms in patients undergoing head CTA in the hospital to be 12%, the total sample size needed is 5784, with 5090 patients having no intracranial aneurysms.

Sample size calculations for noninferiority of specificity with a noninferiority margin of 5%: Using a one-sided *α* level of 0.025 and a power of 0.80 and estimating a specificity of 0.90 in the Sham-AI-aided strategy versus 0.88 in the True-AI-aided strategy, along with accounting for a 5% attrition rate, we determined that a sample size of 3596 patients without intracranial aneurysms is needed to achieve the desired statistical power. According to the sample calculations for the superiority of sensitivity, 5090 patients without intracranial aneurysms will be included, providing a power of 0.928.

**Fig. 4:**
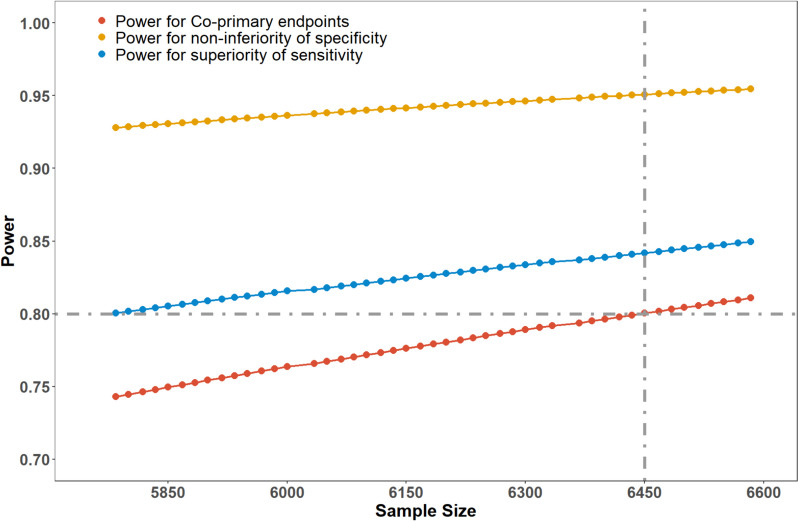
Power calculation of sensitivity, specificity, and general power according to sample size iteration. The combined power for the entire trial is 0.800 (0.841*0.951) when the sample is 6450, in which the power for sensitivity and specificity is 0.841 and 0.951, respectively

A sample size iteration was processed from 5784, and the power for coprimary endpoints was 0.742 (0.80*0.928). When the sample size reached 6450, with 774 patients with intracranial aneurysms and 5676 patients without intracranial aneurysms, the power for superiority of sensitivity was determined to be 0.841, and the power for noninferiority of specificity was 0.951. Then, the power for coprimary endpoints was 0.800 (0.841*0.951) (Fig. 4). Therefore, a total sample size of 6450 is finally determined.

#### Data collection

Data will be collected in a standard case-report form through a web-based electronic data capture (EDC) system and anonymized for further analysis. The data will include baseline information, medical history, lifestyle habits, life quality evaluation, CTA reports, and AI results. Regular quality monitoring and database checking will be performed at each center to ensure data accuracy. In addition, the AI workstation automatically records the reading time for each case in the background. Details of the safety evaluation are provided in the Supplementary Appendix.

#### Data storage and security

All trial data will be securely stored in a dedicated EDC system. This system employs robust security measures, including encryption and access controls, to ensure the confidentiality and integrity of the data. Trial data will be retained for a minimum of 10 years after the completion of the study in accordance with regulatory requirements and sponsor policies. This retention period allows for the preservation of data for potential future analyses, audits, or regulatory inspections. Measures will be implemented to safeguard the confidentiality and privacy of participant data, including de-identification of personal identifiers and compliance with applicable data protection laws and regulations.

#### Data analysis plan

The intention-to-treat (ITT) population will include all patients who are randomized while exclude those who withdrew consent; modified ITT (mITT) population will exclude those whose CTA are not interpreted by the study radiologists, and those with the corresponding AI process failing, and those who withdrew consent; while the Per-protocol (PP) population will only include patients who receive the randomized treatment and do not exhibit major protocol violations, excluding (1) those who withdrew consent; (2) those whose CTA are not interpreted by the study radiologists; (3) those with the corresponding AI process failing; (4) those with the participating radiologists chose not to review AI suggestions, which will be recorded by the background record.

The hypothesis of the study is that the patient-level sensitivity in the intervention arm is superior to that in the control arm, and the patient-level specificity is noninferiority, with a noninferiority margin of 5%. A comparison between the experimental group and the control group for the two primary outcomes will be conducted using the chi-square test or Fisher’s exact test. The results will include the *p* value (2-sided) and the ratio of patient-level sensitivity and specificity. The primary analysis will be based on the ITT population.

Continuous variables will be reported as the means with standard deviations (SDs) or medians with interquartile ranges (IQRs), depending on their distributions. Categorical variables will be presented as numbers and percentages. Statistical significance was set at *p* < 0.05. A 95% CI will be calculated using the Clopper-Pearson method.

The following subgroups will be included:

- Centers (25 centers), provinces (10 provinces), geographical areas and physician working experiences (working experiences of < 10 years vs. ≥ 10 years) and physician level (junior radiologists vs senior radiologists).
- Size (< 5 mm vs. ≥ 5 mm) and locations of intracranial aneurysms (anterior vs posterior, internal carotid artery vs. middle cerebral artery vs. anterior cerebral artery vs. posterior communication artery vs. anterior communication artery vs. vertebral basilar artery vs. others).
- Gender of patients (male vs. female), age (≤ 54 years or. > 54 years), SAH status (with vs. without SAH).
- History of head DSA or surgery (yes vs. no).
- Subsequent head DSA or surgery (yes vs. no).

In this study, only participants who have underwent head CTA will be randomized and included in the analysis. Therefore, missing CTA exams are not expected in the trial. The potential missing data include the following: (1) study reports lost: patients whose CTA exams were accidentally interpreted by other radiologists not involved in the trial, or the reports cannot be retrieved due to other reasons. (2) Baseline characteristic information: the missed data will be marked with “NA” and will not be included in the analysis.

#### Dissemination of results

The data in this study are the properties of the principal investigator and the other co-investigators. This publication is the responsibility of the principal investigator. All co-investigators will have access to anonymized trial data for further analysis and publication of peer-reviewed journal articles.

## Discussion

This double-blinded RCT rigorously assessed whether AI can improve radiologists’ efficacy in intracranial aneurysm diagnosis by CTA and patients’ short-term and long-term outcomes in the real world by comparing the True-AI model to the Sham-AI control (acting as a placebo). To the best of our knowledge, this is the first prospective double-blinded AI RCT in radiology. Crucially, we used the Sham-AI model as a placebo to realize “double-blindness” in AI intervention trials within the field of radiology AI studies. This trial design can act as a typical paradigm for future AI RCTs in radiology.

Blinding to the group assignment is essential to mitigate biases (automation bias or Hawthorne effect) when performing RCTs [14–16, 35]. In open-label studies, working in parallel with AI systems can lead clinicians to potentially alter their practice, and they may find themselves under pressure to either surpass AI performance or overly rely on the model, creating challenges in determining the true impact of AI [29, 36]. Only two blinded randomized trials on AI have been published in echocardiography and colonoscopy [22, 37]. In the study of echocardiography, they assessed the effect of the initial assessment by AI versus conventional initial assessment by a sonographer (the active comparator) on the final interpretation of left ventricular ejection fraction by a cardiologist, while the way to mask cardiologists is not a common practice, and no direct comparison of AI with cardiologist assessment is conducted [22]. In the study of gastroenterology, the double-blind aspect relied on the presence of an independent observer to report the location of any visible AI alert box only if it had not been detected by the operating endoscopist [38], while this way of masking cannot be applied in other areas such as radiology interpretation, where 75% of the FDA-cleared AI algorithms target [39].

To date, there is no head-to-head comparison applying sham-control in RCTs evaluating the effect of AI intervention in radiology, and researchers worry that it would raise ethical concerns about increasing overall diagnostic error [40, 41]. Our team proposed a Sham-AI intracranial aneurysm detection model as a placebo comparator in the field of radiology, with a patient-level sensitivity close to zero and a similar specificity to True-AI, so that the Sham-AI model can mimic the True-AI model and would not help radiologists detect additional aneurysms, exposing the radiologists to the same incidental effects of the Standard-AI at the same time. A crossover trial demonstrated that radiologists had a noninferiority patient-level sensitivity and specificity with Sham-AI augmentation (Shi Z, Hu B, Lu MJ, Zhang MT, Yang HT, He B, Ma JY, Hu CF, Lu L, Li S, et al: Propose and validation of a placebo control for AI models in intracranial aneurysms detection: a Multi-centre, Multi-reader, Blinded Crossover Study, unpublished). Therefore, in the current study, by applying Sham-AI, Hawthorne effect could be mitigated; by adopting the mode of AI being the second reader and double-reading, automation bias could be mitigated. Thus, the exact contribution of AI to AI-clinician collaboration could be extracted.

AI methods may be brittle [42], and this study is designed to address several challenges: first, to improve the sensitivity of intracranial aneurysm diagnosis without the compromise of lowering specificity in CTA with the implementation of AI and to mitigate automation bias and Hawthorne effect in both arms; second, to further explore the impact of the AI system on the clinical management, prognosis, and medical costs of patients with/without aneurysms in real healthcare settings, which is more important than diagnosis alone and represents the future of AI in medicine [17].

In addition, we chose intracranial aneurysms as the target lesion not only because of the challenges of diagnosis but also because of continued uncertainty regarding the optimal management of unruptured intracranial aneurysms (preventive aneurysm repair versus observation) [4]. This paradox would be exacerbated by increasing the detection rate of unruptured aneurysms by the introduction of AI models, especially small aneurysms. It is not clear whether more detected unruptured intracranial aneurysms, assisted by AI technology, would favor patient prognosis, and whether increased detection would increase unnecessary preventive aneurysm repair, or false-positive and small or tiny aneurysms cause patient anxiety and depression in downstream management. Therefore, the target patients are those with mRS ≤ 3 who can participant in questionnaire inquiry and unlikely harboring ruptured aneurysms, to explore the impact of AI on intracranial aneurysms, and highlight the real-world influence of AI on clinical practice, especially in the field of interpretation of medical images.

We plan to enrol 6450 participants in more than 10 provinces across China to represent the influence of AI in clinical practice at a national level. The study is the first prospective double-blinded RCT of AI in radiology and will provide the highest-level evidence for the application of AI systems in clinical settings, not limited to intracranial aneurysms. It is highly expected that the study may set a typical paradigm for AI studies in the radiology field.

### Trial status

The enrollment of this study is not yet initiated at the time of manuscript submission.

Current protocol version: 05 (15/05/2024).

Recruitment would be started on May, 2024.

Expected date for ending recruitment: May, 2025.

### Roles and responsibilities

The principal investigator and research physician contributed to the following aspects: designing and conducting the trial, preparing the protocol and revisions, and publishing the study reports.

### Supplementary Information

Supplementary Material 1.

Supplementary Material 2.

## Data Availability

All principal investigators will be given access to the cleaned datasets. All datasets will be password protected. Project principal investigators will have direct access to their own site datasets and will have access to other site data by request. The data in this study are available from the corresponding author upon reasonable request. The study protocol is accessible in this manuscript and on the registration website. The statistical software is publicly available.
