# Supplementary material for: Assessing the Impact of an Artificial Intelligence-Based Model for Intracranial Aneurysm Detection in CT Angiography on Patient Diagnosis and Outcomes (IDEAL Study)—a protocol for a multicenter, double-blinded randomized controlled trial
Source: Trials. 2024 Jun 4;25:358. doi: 10.1186/s13063-024-08184-9 (PMC11151720; doi:10.1186/s13063-024-08184-9)
Supplement: Supplementary file 1 — Supplementary Material 1. [file 13063_2024_8184_MOESM1_ESM.docx]

Supplementary Appendix

This appendix has been provided by the authors to give readers additional information about the work.

**Supplementary Appendix**

**Table of Contents**

**Supplementary methods - Recruited cases and study withdraw** ................................................2

**Supplementary methods - Requirements for the participating radiologists**..............................2

**Supplementary methods - Adherence and protocol deviations** ...................................................3

**Supplementary methods - Study monitoring** ................................................................................3

**Fig. S1. Web-based workstation for intracranial aneurysm diagnosis**........................................4

**Fig. S2.** **Overview of the architectures of True-AI and Sham-AI models**.......................................4

**Table. S1.** **Overview of illustration of the second outcomes**........................................................................5

**Supplementary methods - Cases recruitment and study withdraw**

In each participating centre a lead investigator will be identified as Local PI, and is responsible for all aspects of local organisation, including identification, recruitment, data collection and completion of CRFs, along with follow up of study patients and adherence to study protocol and investigators brochure. Local staff members will be pretrained for identifying potential recruits and taking consent before the examination. Consecutive patients will be informed about the study and assessed for eligibility. Patients eligible to participate in this study will be provided with further discussions and informed consent. Discussions may be completed by a local staff member. Written informed consent will be obtained from all participants who agree and wish to take part in the study.

Participants can discontinue their participation in the study at any time without any consequence to their continued head CTA examination, interpretation, work up, and treatment. Depending on when the subject chooses to discontinue his/her participation, different actions will be applied. If head CTA exam has not yet been conducted, the exam will be classified as a non-participant in the Picture Archiving and Communication Systems (PACS) and the case will be referred to conventional reading procedure. If head CTA exam has already been conducted and without regard to interpretation status, the participants will be excluded from further study analyses. Information about these participants will be stored on a separate file.

**Supplementary methods - Requirements for the participating radiologists**

The participating radiologists are required to meet the following criteria: the junior radiologist should have a working experience more than 3 years in head CTA interpretation; and the senior radiologists should be dedicated neuroradiologists who are qualified to review the radiology reports from a junior radiologist and have worked for more than 8 years, and have reviewed more than 100 head CTA exams in the past 1 year at their local site, which is determined by the Steering Committee. Once a patient is enrolled, the patient’s report task will be transformed to the two radiologists. The final diagnosis will be extracted from the final radiology report. The reference standard for all examinations will be determined by the Core Image Center.

**Supplementary methods -** **Adherence and protocol** **deviations**

To enhance the validity of the data, face-to-face adherence reminder sessions and a pilot study will be conducted before enrollment at each study site. Additionally, a key method will be followed for assessing the adherence. The AI workstation can automatically record the timepoints of starting reading and end reading for each case in the background to monitor whether the participating radiologists review AI results, and the adherence assessments will be assessed.

The following conditions will be defined as protocol deviations: (1) those whose CTA are not interpreted by the study radiologists; (2) those with the corresponding AI process failing; (3) those with the participating radiologists chose not to review AI suggestions, which will be recorded by the background record.

**Supplementary methods - Study monitoring**

Research assistants of chief investigating center are responsible for regular study monitoring. A Data Monitoring Committee (DMC) has been established. The DMC is independent of the study organizers.

DMC and Quality Control and Inquiry Committee will meet at least once a month to monitor the progress of all aspects of the trial and ensure that the trial meets the highest standards of ethics and patient safety. The members may suggest trial amendments regarding the safety of patients or early trial termination, but the final decision rests with the steering committee. Members of the data monitoring committee will not participate in the trial. Auditing is performed by exploring the trial dataset or performing site visits regularly for each center.

**References**

1. National Cancer Institute. Common terminology criteria for adverse events. UpToDate Waltham, MA: UpToDate. 2013:1-9


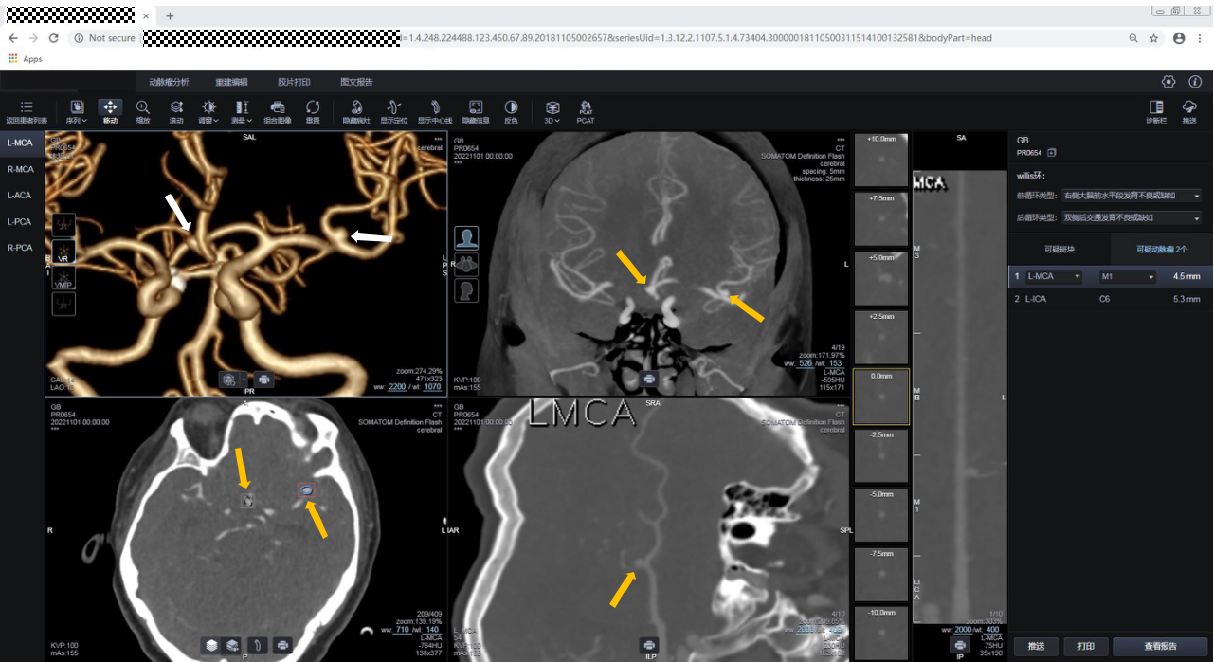


**Fig. S1. Web-based workstation for intracranial aneurysm diagnosis.**

This patient harbored two intracranial aneurysms located at the left middle cerebral artery and anterior communicating artery, and True-AI model indicated the two intracranial aneurysms (arrow).


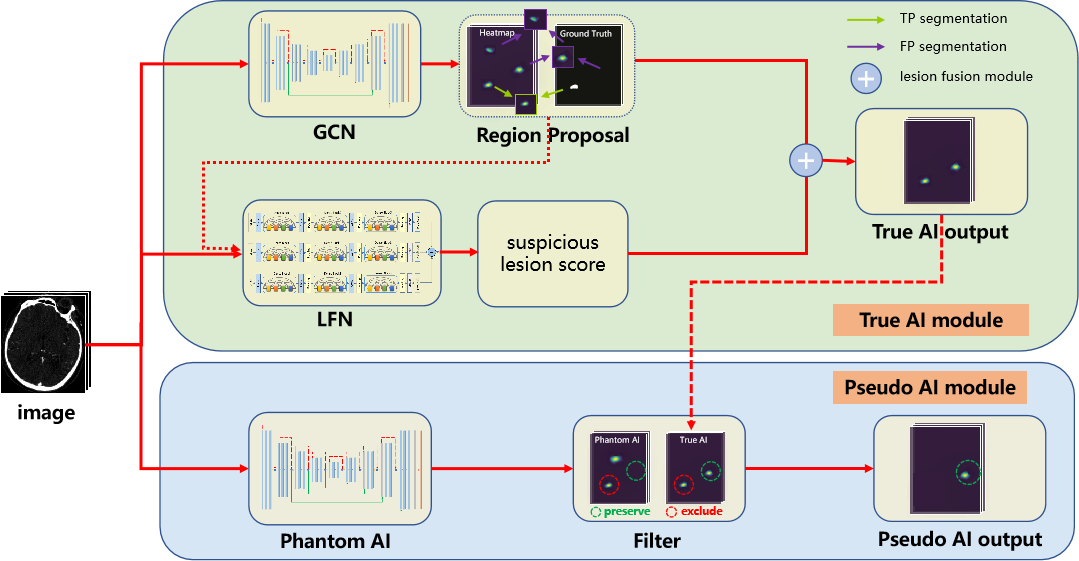


**Fig****. S2 Overview of the architectures of True-AI and Sham-AI models [23].**

The True-AI model is a fusion module that combines the outputs of the GCN and LFN modules and filters false positive lesions, yielding the final detection (top part). The Sham-AI model is created by removing the Phantom-AI detections from the True-AI results (bottom). AI = artificial intelligence, FP = false positive, GCN = global context network, LFN = local fine-grained network, TP = true positive.

**Table. S1.** **Overview of illustration of the second outcomes**

|  | **Secondary outcomes** | **Domain** | **Measure** | **Metric** | **Method of aggregation** | **Timepoint** |
| --- | --- | --- | --- | --- | --- | --- |
| 1 | Differences of other diagnostic performance metrics for intracranial aneurysms | Qualitative data | **Accuracy**: the proportion of patients with true-positive and true-negative diagnosed aneurysms among the total number of patients in each arm.  **Lesion-level sensitivity**: the proportion of true-positive diagnosed aneurysms among the total number of reference standard aneurysms in each arm.  **Positive predictive value (PPV)**: the proportion of patients with true-positive diagnosed aneurysms among patients with true-positive and and false-positive diagnosed aneurysms.  **Negative predictive value (NPV)**: the proportion of patients with true-negative diagnosed aneurysms among patients with true-negative and and false-negative diagnosed aneurysms. | Comparing the values between the two arms | Proportion | When the enrollment is finished |
| 2 | Differences of the diagnostic performances for other intracranial lesions | Qualitative data | **Accuracy**: the proportion of patients with true-positive and true-negative diagnosed intracranial arterial stenosis (≥ 50%)/occlusion/intracranial tumors among the total number of patients in each arm.  **Lesion-level sensitivity**: the proportion of true-positive diagnosed intracranial arterial stenosis (≥ 50%)/occlusion/intracranial tumors among the total number of reference standard intracranial arterial stenosis (≥ 50%)/occlusion/intracranial tumors in each arm.  **Positive predictive value (PPV)**: the proportion of patients with true-positive diagnosed intracranial arterial stenosis (≥ 50%)/occlusion/intracranial tumors among patients with true-positive and and false-positive diagnosed intracranial arterial stenosis (≥ 50%)/occlusion/intracranial tumors.  **Negative predictive value (NPV)**: the proportion of patients with true-negative diagnosed intracranial arterial stenosis (≥ 50%)/occlusion/intracranial tumors among patients with true-negative and and false-negative diagnosed intracranial arterial stenosis (≥ 50%)/occlusion/intracranial tumors. | Comparing the values between the two arms | Proportion | When the enrollment is finished |
| 3 | Differences of the detection rates of intracranial lesions according to radiology reports | Qualitative data | **Detection rates**: the proportion of patients with report-positive diagnosed aneurysms/intracranial arterial stenosis (≥ 50%)/occlusion/intracranial tumors among the total number of patients in each arm. | Comparing the values between the two arms | Proportion | When the enrollment is finished |
| 4 | Differences of the workload of head CTA interpretation | Measurement data | Time (seconds) of interpreting head CTA images, the number of consensus meetings (times) | Comparing the values between the two arms | Mean/median^🞷^ | When the enrollment is finished |
| 5 | Differences in the proportion of participants with resource use between True-AI and Sham-AI group | Measurement data | The number of care encounters (in person) during follow-up, the number of care encounters (in person) for aneurysms during follow-up, the total number of cerebral artery tests (including DSA, CTA, MRA and high-resolution vessel wall MR imaging). | Comparing the values between the two arms | Mean/median^🞷^ | At the 3-month and 12-month follow-ups |
| 6 | Differences in the proportion of participants with treatment-related clinical events between True-AI and Sham-AI group | Qualitative data | The number of clinical follow-ups, number of subsequent hospitalizations, number of hospitalization for intracranial aneurysms, number of patients with morbidity with modified RS ≥ 3 due to intracranial hemorrhage or treatment, number of patients undergoing DSA, number of patients with in-hospital mortality, number of patients with different methods for aneurysm treatment (conservative/coil/clip/others), number of patients with aneurysm treatment-related complications (intraoperative rupture, death, stroke, etc.), number of patients with recurrence or residual intracranial aneurysm after surgery | Comparing the values between the two arms | Proportion | At the 3-month and 12-month follow-ups |
| 7 | Differences in the proportion of participants with treatment-related clinical events between True-AI and Sham-AI group | Measurement data | Length of hospital stay, detection rate of intracranial aneurysms among DSAs, detection rate of no abnormality among DSAs | Comparing the values between the two arms | Mean/median | At the 3-month and 12-month follow-ups |
| 8 | Differences of life quality evaluation | Measurement data | EuroQol 5-Dimensional (EQ-5D-5L) scores, shift work assessment, Pittsburgh Sleep Quality Index (PSQI), Patient Health Questionnaire-9 (PHQ-9), Hospital Anxiety and Depression Scale (HADS), Short-Form 36 Health Survey (SF-36), Modified Rankin Scale (mRS) score | Comparing the values between the two arms | Mean/median | At the 3-month and 12-month follow-ups |
| 9 | Differences in the proportion of participants with outcomes of aneurysm-related events | Qualitative data | The number of patients with all-cause mortality, mortality of aneurysm rupture, the number of patients with aneurysm growth, aneurysm rupture, subarachnoid hemorrhage, and stroke (hemorrhagic stroke, ischemic stroke) | Comparing the values between the two arms | Proportion | At the 3-month and 12-month follow-ups |

CTA = CT angiography, DSA = digital subtraction angiograms, MRA = magnetic resonance angiography.
